# Carbon Nanoparticles Enhance Drought Tolerance Through the Improvement of Morphological and Physiological Traits in Maize Hybrids

**DOI:** 10.3390/plants15081185

**Published:** 2026-04-12

**Authors:** Jiovana Kamila Vilas Boas, Fábio Steiner, Gilciany Ribeiro Soares, Jorge González Aguilera, Alan Mario Zuffo, Ofelda Peñuelas-Rubio, Leandris Argentel-Martínez, Ugur Azizoglu

**Affiliations:** 1 Department of Crop Science, State University of Mato Grosso do Sul (UEMS), Aquidauana 79200-000, Mato Grosso do Sul, Brazil; jiovanakv.21@gmail.com (J.K.V.B.); gilribeiro1940@hotmail.com (G.R.S.); 2 Department of Agronomy, State University of Mato Grosso do Sul (UEMS), Cassilandia 79540-000, Mato Grosso do Sul, Brazil; jorge.aguilera@uems.br; 3 Department of Agronomy, State University of Maranhão (UEMA), Balsas 65800-000, Maranhão, Brazil; alanzuffo@professor.uema.br; 4 Yaqui Valley Technological Institute, National Technology of Mexico (TecNM), Bacum 85276, Sonora, Mexico; ofeperub@gmail.com (O.P.-R.); oleinismora@gmail.com (L.A.-M.); 5 Department of Crop and Animal Production, Safiye Cikrikcioglu Vocational College, Kayseri University, Talas 38280, Kayseri, Türkiye; azizogluugur@hotmail.com; 6 Genome and Stem Cell Center (GENKOK), Erciyes University, Melikgazi 38280, Kayseri, Türkiye

**Keywords:** carbon nanoparticles, nanofertilizer, drought stress, water use efficiency, *Zea mays*

## Abstract

Drought stress severely limits maize growth and productivity worldwide. In this study, we examined the effects of foliar-applied carbon nanoparticles (CNPs) on morphological and physiological traits in maize plants exposed to drought stress for 25 days. Two maize hybrids, one drought-tolerant (LG 36745 PRO4) and one drought-sensitive (AG 8088 PRO2), were fertilized with 0 or 1.0 mL L^−1^ of a CNP-based nanofertilizer at the V_2_ growth stage and exposed to three drought levels: 0 MPa (control), −0.4 MPa (moderate stress), and −0.8 MPa (severe stress). The experiment followed a 2 × 2 × 3 factorial design (hybrid × CNP treatment × drought level) with four replicates. Results indicated that drought stress adversely affected most morphological and physiological traits, particularly in the drought-sensitive hybrid. However, foliar CNP application significantly alleviated the adverse effects of drought in maize plants under moderate and severe stress, primarily by preserving plant water status, enhancing water use efficiency, carboxylation efficiency, photosynthetic rate, and initial growth in challenging environments. These findings will provide the basis for future research on management practices adopted to control drought and ensure the development of modern and sustainable agriculture.

## 1. Introduction

Maize (*Zea mays* L.) is one of the world’s major cereal crops due to its extensive use in food, feed, biofuel, and industrial applications. Although Brazil is one of the world’s largest maize producers, the growth and yield of the crop have frequently been impacted by adverse environmental conditions, such as high temperature and water deficiency [1,2,3]. The Brazilian Cerrado region accounts for nearly two-thirds of national maize production [4]. However, the Cerrado has a pronounced dry season in winter (April to September), leading farmers in many cases to sow maize under suboptimal soil moisture, compromising the seed imbibition process and early plant establishment in the field [3].

The lower maize plant population compromises the development and yield potential of the crop [5,6,7]. Low soil-water availability also triggers various morphological, physiological, and metabolic changes in plants [6,8,9], inhibiting leaf expansion, photosynthesis, transpiration, and nutrient uptake. Thus, drought stress presents significant challenges to global maize production, making research into innovative, sustainable mitigation strategies crucial for advancing food security, plant protection, and environmental resilience amid climate change [10,11].

Nanofertilizers represent an innovative and sustainable approach to enhancing plant growth, physiology, and metabolism under drought conditions [12,13,14,15,16]. However, their efficacy in mitigating drought’s negative effects hinges on the plant’s tolerance mechanisms, genotype-specific tolerance, stress severity, and interactions among genotype, nanofertilizer, and environment. Key drought tolerance mechanisms in early maize growth involve greater photoassimilate allocation to roots, chlorophyll maintenance, leaf area reduction, and synthesis of metabolites for osmotic adjustment and turgor preservation [3,6,17]. These mechanisms collectively support optimal plant development in adverse conditions.

Among nanomaterials, carbon nanoparticles (CNPs) have emerged as promising tools in sustainable agriculture due to their high surface reactivity and multifunctional roles in soil remediation and plant stress tolerance [18]. CNP-based nanofertilizers offer a sustainable option for alleviating drought stress in agricultural systems, as their molecules are biodegradable and environmentally benign. CNPs also possess unique structural and mechanical properties, and their use in drought management has expanded recently [19,20,21,22]. CNP application alleviated drought stress by regulating water status, boosting chlorophyll and photosynthetic rates, and reducing oxidative stress via antioxidant activation and ROS scavenging [21]. Foliar CNP application similarly mitigated drought damage to growth, metabolism, and physiology in bell pepper (*Capsicum annuum* L.) plants [22]. Shekhawat et al. [19] reported that CNP application enhances the antioxidant defense and abiotic stress tolerance in cowpea (*Vigna radiata* L. Wilczek) plants. Similarly, CNP application reduced drought’s adverse effects on tomato (*Solanum lycopersicum* L.) growth and yield, particularly through improved water status, use efficiency, and antioxidant regulation [20].

Thus, CNPs present an excellent tool for enhancing growth, nutrition, biochemistry, and physiology in plants under stress. This approach can lessen drought impacts, lower production costs, and promote sustainable, innovative farming systems. However, the effects of CNP-based nanofertilizers like Arbolin Biogenesis on inducing drought tolerance in maize remain unexplored. This study, therefore, assessed the effects of foliar CNP application on morphological and physiological traits in maize under varying drought levels. We evaluated CNP efficacy in enhancing drought tolerance in two common Cerrado maize hybrids: one tolerant and one sensitive.

## 2. Results

Analysis of variance revealed significant effects (*p* ≤ 0.05) from hybrids, drought levels, and CNP application (Table 1). A significant interaction between hybrid, drought level, and CNP application (H × D × CNP) was observed for most measured traits (*p* ≤ 0.05). Significant hybrid × drought and hybrid × CNPs interactions for many traits indicate differential responses between tolerant and sensitive hybrids under stress or CNP treatment. Accordingly, we unfolded interactions and present responses for each hybrid separately.

Coefficients of variation (CVs) were <10% for all traits, indicating high precision for lab assays (Table 1). The CV is a measure of dispersion used to estimate the precision of the experimental trial, and, when the values are less than 10%, it indicates that the results of the experiment have excellent precision.

### 2.1. Morphophysiological Responses Compared Among Maize Hybrids

**Figure 1:**
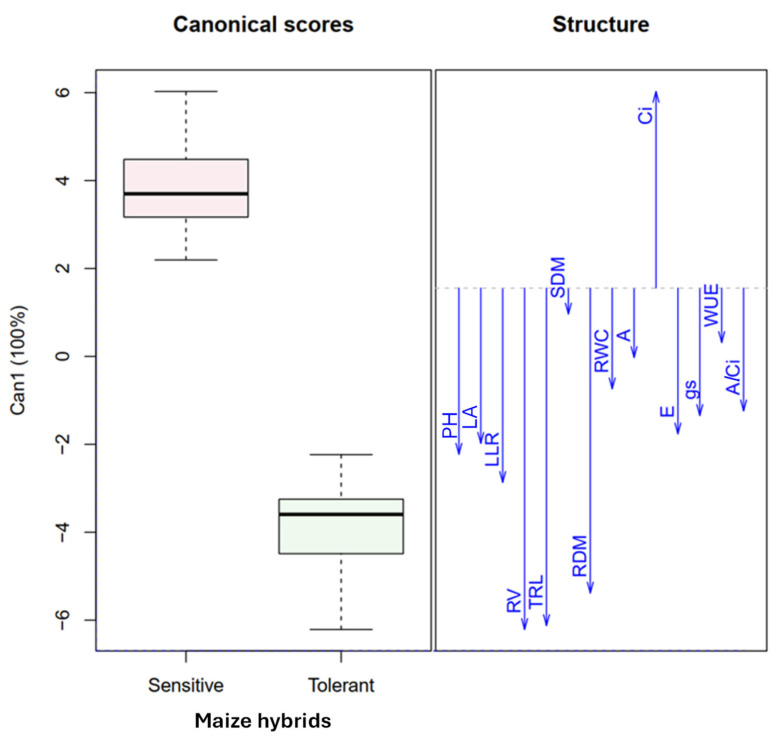
Canonical analysis between the maize hybrids and plant morphophysiological traits. Abbreviations: PH: plant height. LA: leaf area. LLR: length of the longest root. TRL: total root system length. RV: root volume. SDM: shoot dry matter. RDM: root dry matter. RWC: relative water content. *A*: photosynthetic rate. Ci: intercellular CO_2_ concentration. *E*: transpiration rate. *g*_S_: stomatal conductance. WUE: water use efficiency. *A*/Ci: carboxylation efficiency.

The maize hybrids exhibit distinct morphophysiological responses when exposed to drought stress conditions (Figure 1). Drought-tolerant maize hybrid plants have greater root length, volume, and dry matter compared to drought-sensitive hybrids, allowing for greater water absorption, maintenance of relative leaf water content, and improved physiological metabolism (*A*, *E*, *g*_S_, WUE, and *A*/Ci) of the plants. Drought-tolerant hybrid maintains a larger leaf area and higher chlorophyll content, which improves photosynthetic activity and shoot dry matter production. In turn, drought-sensitive maize hybrid plants have a higher internal CO_2_ concentration, indicating the impairment of the plant’s physiological activity and the greater maintenance of CO_2_ within the leaves under drought conditions.

### 2.2. Effects of Foliar CNP Application on Maize Morphological Traits

**Figure 2:**
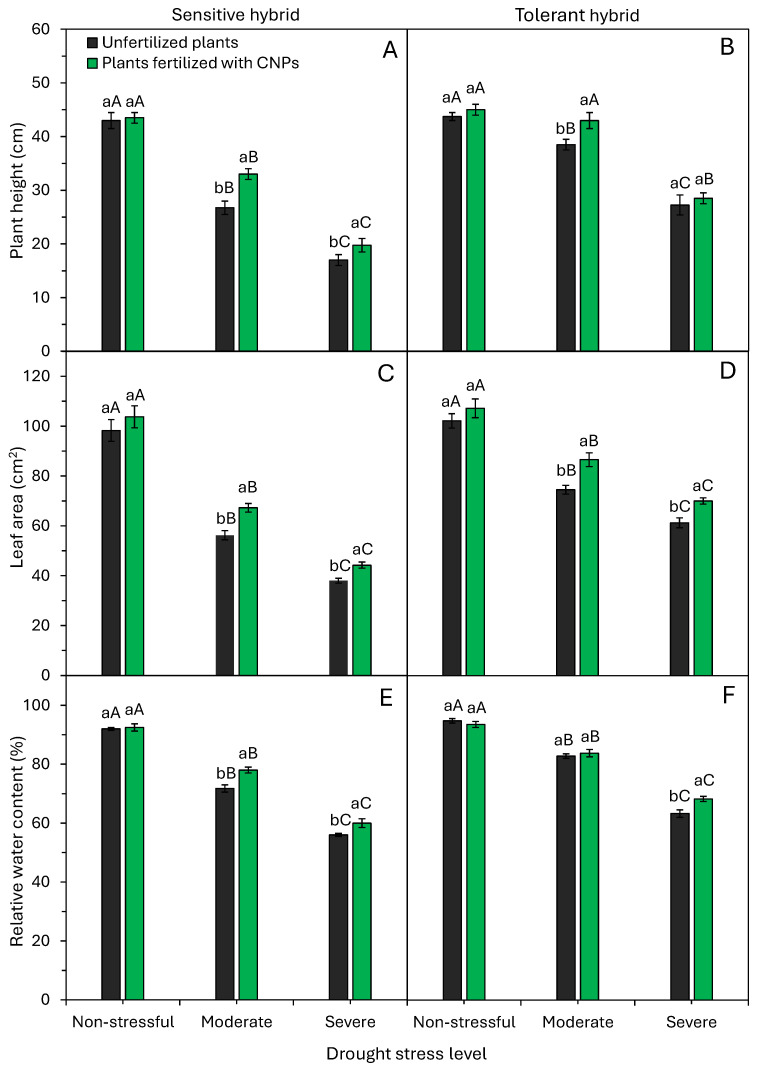
Effect of drought stress levels and foliar application of carbon nanoparticles (CNPs) on plant height (**A**,**B**), leaf area (**C**,**D**), and relative water content (**E**,**F**) of maize plants from a drought-sensitive hybrid (**A**,**C**,**E**) and another drought-tolerant hybrid (**B**,**D**,**F**). Bars followed by distinct lowercase letters for the foliar application of CNPs or distinct uppercase letters for the drought stress levels show significant differences by the Tukey test (α = 0.05). Data refers to mean values (*n* = 20) ± standard error of the mean.

Drought stress levels and CNP application significantly (*p* < 0.05) influenced plant height, leaf area, and relative water content of maize hybrids (Figure 2). Drought levels limit shoot growth in both maize hybrids; however, the greatest impact of drought stress on plant height is observed in the drought-sensitive hybrid (Figure 2A,B). Under severe stress, sensitive and tolerant hybrids exhibited average height reductions of 57% (43.2 to 18.5 cm) and 37% (44.4 to 27.9 cm), respectively, compared to controls. Foliar CNP application, however, mitigated these height reductions. CNP-treated sensitive hybrid plants had greater heights under moderate and severe stress (Figure 2A). Tolerant hybrid plants similarly showed height increases with CNPs under moderate stress (Figure 2B).

Leaf area decreased progressively with increasing drought intensity in both hybrids (Figure 2C,D). CNP fertilization, however, yielded significantly larger leaf areas under moderate and severe stress. Under non-stress, relative water content stayed consistent at 92–95% for both hybrids (Figure 2E,F). Drought reduced this to 72–84% under moderate stress and 56–68% under severe stress. CNP application attenuated these reductions in the sensitive hybrid under both stress levels and in the tolerant hybrid under severe stress (Figure 2E,F).

**Figure 3:**
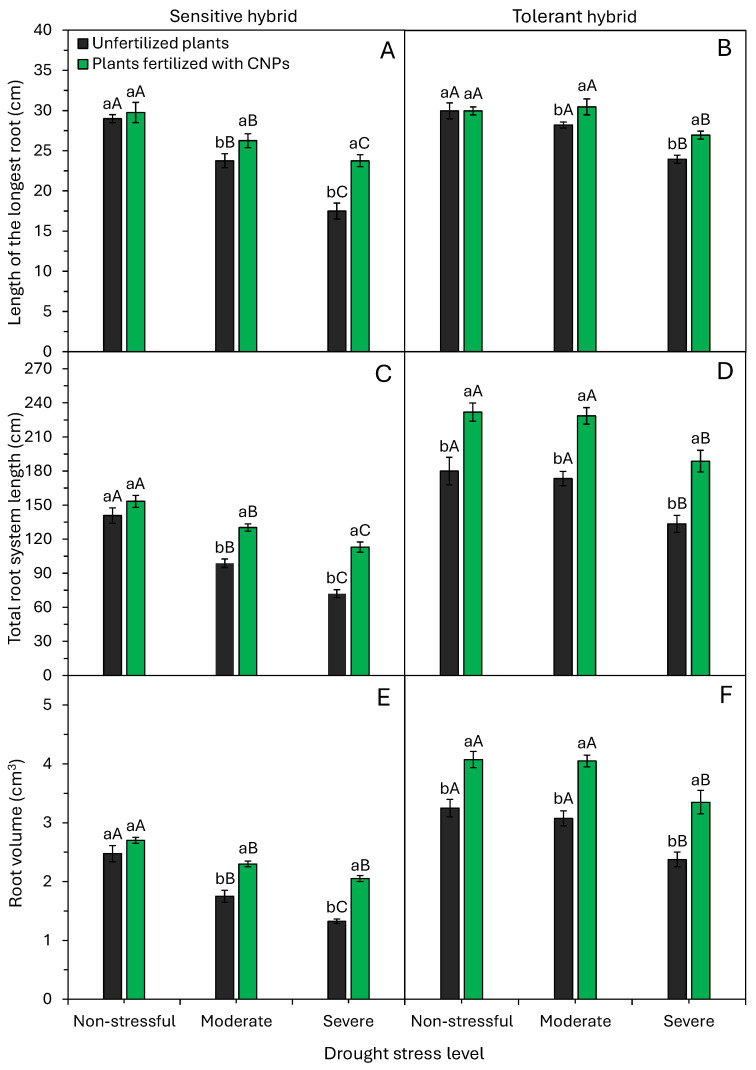
Effect of drought stress levels and foliar application of carbon nanoparticles (CNPs) on the length of the longest root (**A**,**B**), total root system length (**C**,**D**), and root volume (**E**,**F**) of maize plants from a drought-sensitive hybrid (**A**,**C**,**E**) and another drought-tolerant hybrid (**B**,**D**,**F**). Bars followed by distinct lowercase letters for the foliar application of CNPs or distinct uppercase letters for the drought stress levels show significant differences by the Tukey test (α = 0.05). Data refers to mean values (*n* = 20) ± standard error of the mean.

Drought and CNP application significantly (*p* < 0.05) influenced root system growth in both hybrids (Figure 3). Moderate and severe stress inhibited root development in the sensitive hybrid. Longest root length, total root length, and root volume declined progressively with stress intensity (Figure 3A,C,E). In the tolerant hybrid, root growth was inhibited only under severe stress (Figure 3B,D,F).

CNP application mitigated these effects in both hybrids. CNP-treated sensitive plants had longer roots, total roots, and higher volumes under moderate and severe stress (Figure 3A,C,E). Tolerant plants showed enhanced root growth with CNPs under all conditions, except for the longest root length in non-stress (Figure 3B,D,F).

**Figure 4:**
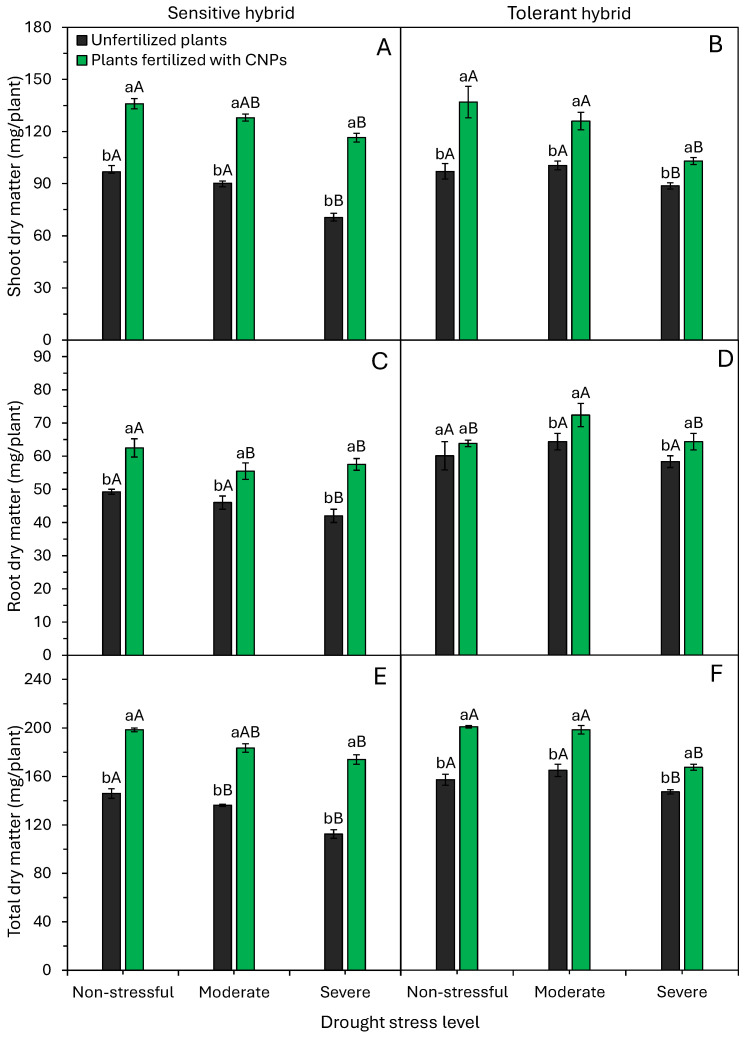
Effect of drought stress levels and foliar application of carbon nanoparticles (CNPs) on shoot dry matter (**A**,**B**), root dry matter (**C**,**D**), and total dry matter (**E**,**F**) of maize plants from a drought-sensitive hybrid (**A**,**C**,**E**) and another drought-tolerant hybrid (**B**,**D**,**F**). Bars followed by distinct lowercase letters for the foliar application of CNPs or distinct uppercase letters for the drought stress levels show significant differences by the Tukey test (α = 0.05). Data refers to mean values (*n* = 20) ± standard error of the mean.

**Figure 5:**
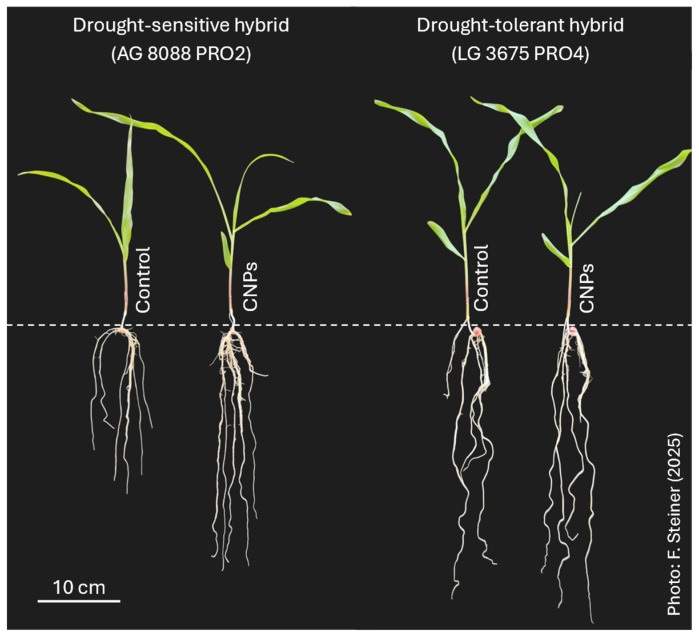
Drought-sensitive maize hybrid (AG 8088 PRO2) and drought-tolerant hybrid (LG 3675 PRO4) plants fertilized with carbon nanoparticles (CNPs) and grown for 25 days under severe drought stress conditions simulated with −0.80 MPa PEG-6000 osmotic solution.

Drought and CNPs significantly (*p* < 0.05) affected shoot, root, and total dry matter accumulation in both hybrids (Figure 4). Severe stress reduced shoot, root, and total dry matter compared to untreated stressed plants (Figure 4). CNPs increased these biomasses in both hybrids versus controls, except for root dry matter in non-stress (Figure 4D). Overall, CNPs enhanced shoot and root growth under drought, particularly in the sensitive hybrid (Figure 5).

### 2.3. Effects of Foliar CNP Application on Maize Physiological Traits

**Figure 6:**
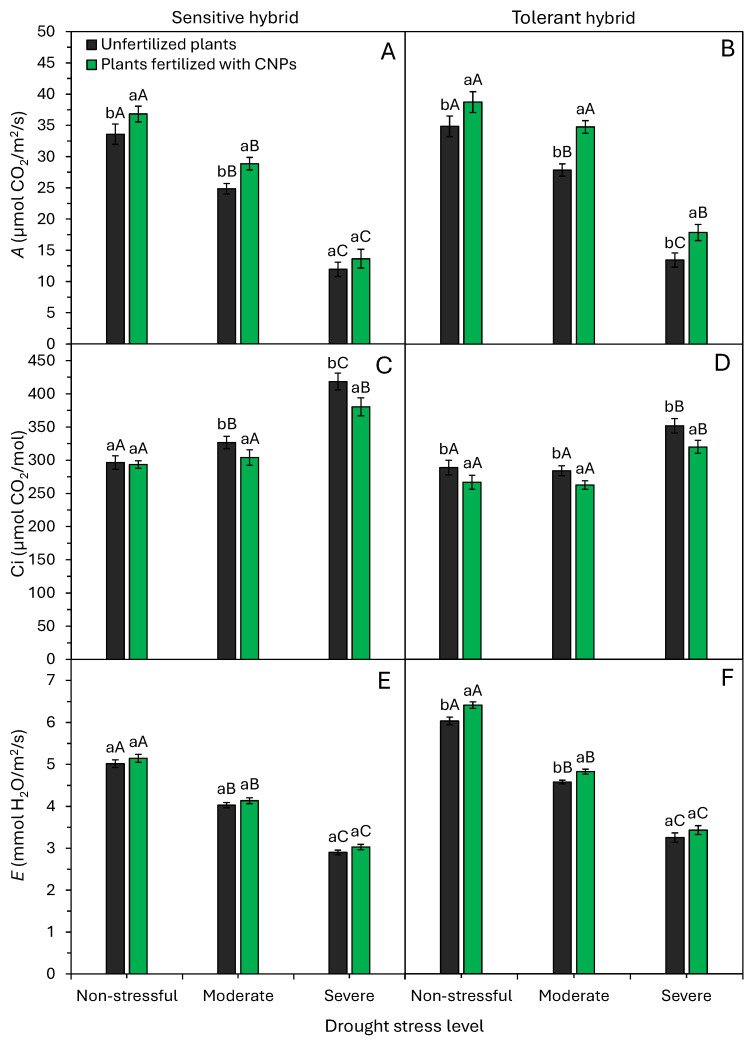
Effect of drought stress levels and foliar application of carbon nanoparticles (CNPs) on the photosynthetic rate—A (**A**,**B**), intercellular CO_2_ concentration—Ci (**C**,**D**), and transpiration rate—E (**E**,**F**) of maize plants from a drought-sensitive hybrid (**A**,**C**,**E**) and another drought-tolerant hybrid (**B**,**D**,**F**). Bars followed by distinct lowercase letters for the foliar application of CNPs or distinct uppercase letters for the drought stress levels show significant differences by the Tukey test (α = 0.05). Data refers to mean values (*n* = 20) ± standard error of the mean.

Drought and CNPs significantly (*p* < 0.05) affected photosynthetic rate, intercellular CO_2_ concentration, and transpiration rate (Figure 6). Photosynthetic and transpiration rates declined progressively with stress intensity in both hybrids. Severe stress reduced photosynthetic rates by 64% (35.2 to 12.8 μmol CO_2_ m^−2^ s^−1^) in sensitive and 57% (36.8 to 15.7 μmol CO_2_ m^−2^ s^−1^) in tolerant hybrids versus controls (Figure 6A,B). Transpiration rates dropped by 41% (5.1 to 3.0 mmol H_2_O m^−2^ s^−1^) and 46% (6.2 to 3.3 mmol H_2_O m^−2^ s^−1^), respectively (Figure 6E,F). CNPs, however, improved photosynthetic rates. Sensitive plants had higher rates with CNPs under non-stress and moderate stress (Figure 6A). Tolerant plants showed increases under all conditions (Figure 6B). CNPs did not affect transpiration in sensitive plants (Figure 6E). Tolerant plants had higher rates with CNPs under non-stress and moderate conditions (Figure 6F).

The intercellular CO_2_ concentration of both maize hybrids was significantly higher in plants grown under severe stress compared to plants grown under moderate or non-stressful conditions (Figure 6C,D). Foliar application of CNPs resulted in the lowest intercellular CO_2_ concentration in maize plants, except for the sensitive hybrid under non-stress conditions (Figure 6C,D).

**Figure 7:**
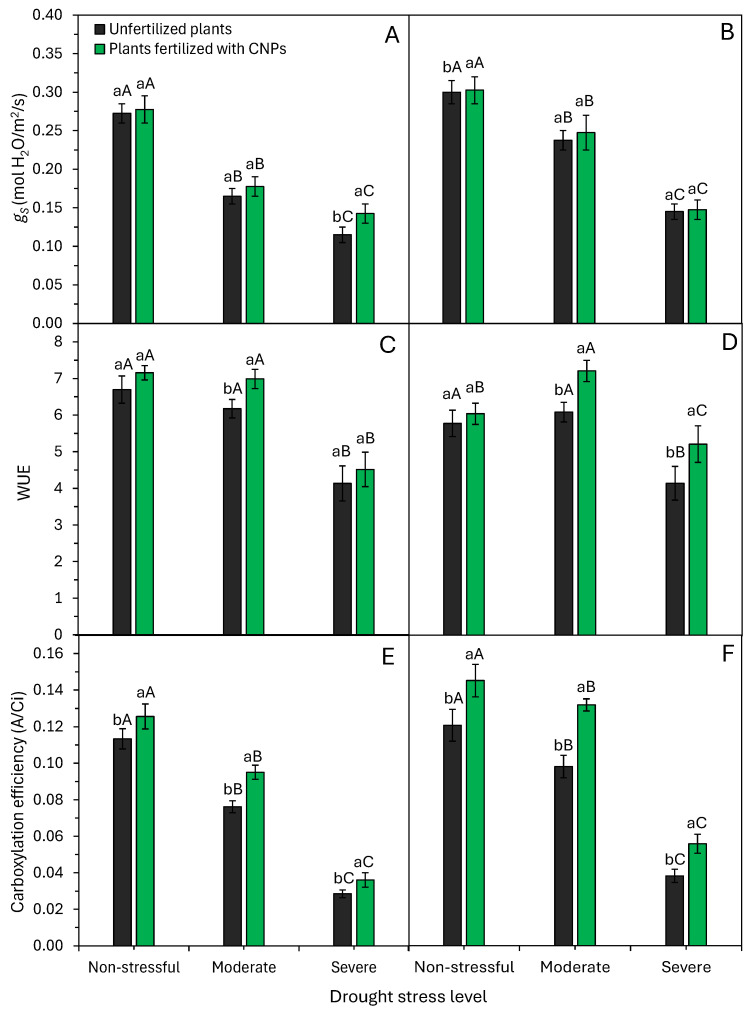
Effect of drought stress levels and foliar application of carbon nanoparticles (CNPs) on the stomatal conductance—*g*_s_ (**A**,**B**), water use efficiency—WUE (**C**,**D**), and carboxylation efficiency—A/Ci (**E**,**F**) of maize plants from a drought-sensitive hybrid (**A**,**C**,**E**) and another drought-tolerant hybrid (**B**,**D**,**F**). Bars followed by distinct lowercase letters for the foliar application of CNPs or distinct uppercase letters for the drought stress levels show significant differences by the Tukey test (α = 0.05). Data refers to mean values (*n* = 20) ± standard error of the mean.

Drought and CNPs significantly (*p* < 0.05) influenced stomatal conductance, water use efficiency, and carboxylation efficiency (Figure 7). These parameters declined progressively with stress intensity in both hybrids. Severe stress reduced stomatal conductance by 54% (0.28 to 0.13 mol H_2_O m^−2^ s^−1^) in sensitive and 50% (0.30 to 0.15 mol H_2_O m^−2^ s^−1^) in tolerant hybrids (Figure 7A,B). Water use efficiency fell by 38% (6.9 to 4.3 μmol CO_2_ mol^−1^ H_2_O) and 26% (5.9 to 4.7 μmol CO_2_ mol^−1^ H_2_O), respectively (Figure 7C,D). Carboxylation efficiency decreased by 75% (0.12 to 0.03) and 62% (0.13 to 0.05), respectively (Figure 7E,F).

Drought, particularly severe, drastically lowered most physiological traits (Figure 6 and Figure 7). Severe stress reduced *A* by 61%, *E* by 45%, gs by 52%, WUE by 38%, and *A*/Ci by 69% versus non-stress. These impairments in *A*, WUE, and *A*/Ci led to reduced growth and dry mass, especially in sensitive hybrids (Figure 4 and Figure 5). Moderate stress decreased shoot and root dry mass by ~5% and 19%, and severe stress decreased them by 5% and 11%, respectively, versus non-stress (Figure 4).

## 3. Discussion

The maize hybrids used in this research were selected by Vilas Boas et al. [3]. The morphological responses of these two hybrids were the most contrasting among the 15 modern Brazilian hybrids exposed to drought stress conditions. Comparative research between drought-tolerant and drought-sensitive hybrids is crucial for modern, sustainable agriculture [17]. This research provides the scientific basis for the development of resilient crops, allowing the optimization of water use and ensuring food security in the face of climate change.

Drought inhibited maize shoot growth (Figure 2 and Figure 4). However, inhibition levels varied between hybrids. Sensitive plants under severe stress had reductions of 57% in height, 59% in leaf area, and 22% in shoot dry matter compared to plants under non-stressful conditions (control) (Figure 2A,C and Figure 4A). Tolerant hybrid plants exposed to severe stress showed 37%, 36%, and 16% reductions, respectively (Figure 2B,D and Figure 4B). These findings indicate drought inhibited elongation and photoassimilate accumulation in both hybrids. A lower rate of plant elongation and dry matter accumulation under drought conditions has been reported in different maize hybrids [3,5].

Reduced growth under drought stress occurs due to disruptions in the cell cycle machinery [23]. Drought perception triggers signaling that activates checkpoints, impairing G1-S transition, slowing DNA replication, or delaying mitosis [24,25], thereby prolonging the cell cycle and restricting growth and dry matter accumulation. Furthermore, drought stress impairs other primary cell growth parameters, such as wall extensibility and cell turgor [26]. Limited water uptake reduces cell turgor pressure, slowing plant elongation and growth [6]. Maize growth rates dropped from 165 to 56 mm day^−1^ under drought [27]. Therefore, cell expansion, a process highly dependent on plant turgidity, is one of the first processes negatively impacted by drought stress, which limits plant height, leaf area, and dry matter accumulation (Figure 2 and Figure 4).

Lower shoot dry matter production of maize under drought conditions (Figure 4A,B) occurs due to the reduced photosynthetic rate (Figure 6A,B), which limits photoassimilate allocation to shoots [6]. As a result of this effect, there is a reduction in leaf production and leaf area (Figure 2). These serve as tolerance mechanisms to minimize transpiration (water loss) and optimize water use efficiencies. Drought often limits cereal growth, including wheat [28], sorghum [29], and maize [3,6,17]. Therefore, the response mechanisms of plants exposed to drought stress have become a crucial topic of environmental research in drought-prone regions.

Morphophysiological responses of maize plants to drought stress typically included smaller leaf area, enhanced root growth, lower water content, and reduced photosynthesis, transpiration, and stomatal conductance (Figure 2, Figure 3, Figure 6 and Figure 7). These results confirm plant responses commonly reported in other studies under drought stress conditions [5,6,28]. Roots encounter drought first, and their length and architecture critically affect water and nutrient uptake under low moisture [6]. Furthermore, the greater growth of the root system in conditions of low water availability is indicative of the greater drought tolerance of the maize hybrid [3]. Walne et al. [6] showed that drought-tolerant maize hybrids have greater root length growth (35%) when compared to stable hybrids. Under drought, photoassimilates are preferentially routed to roots via vascular tissues [9,30]. This increased allocation of photoassimilates to the roots strengthens the growth of the root system and water uptake in the soil profile. Therefore, current plant breeding programs should select genotypes with longer roots for drought tolerance.

Higher intercellular CO_2_ concentration under severe drought stress relates to partial stomatal closure for water conservation, retaining CO_2_ inside leaves [31]. This intercellular CO_2_ increase, paired with lower stomatal conductance, reduces transpiration (Figure 6). Such effects lower photosynthesis via higher stomatal resistance and reduced carboxylation efficiency [9]. Under low water conditions, this intercellular CO_2_ concentration rise may stem from reduced CO_2_-fixing enzyme activity, impairing water use efficiency [32]. Under non-stressful conditions, plants avoid reducing leaf area or transpiration or raising CO_2_, leading to higher photosynthesis and growth, as reported in this study.

Photosynthesis, central to plant energy, can suffer greatly under drought conditions. Drought curbs photosynthetic efficiency through biochemical and hydric constraints that diminish key photosynthetic components and induce stomatal closure [9]. However, our results showed that foliar application of CNPs plays a key role in mitigating the negative effects of drought on the photosynthetic rate of maize plants (Figure 6). Balanced photosynthesis is vital for growth, biomass, and yield [9,28]. Therefore, the application of CNPs can be used to improve crop development and production under adverse environmental conditions.

Maize plants fertilized with CNPs have higher relative water content, photosynthesis rate, and leaf area, and higher initial growth when compared to control plants (Figure 2, Figure 3, Figure 4 and Figure 6). Bell pepper plants fertilized with CNPs also have higher relative water content, chlorophyll content, photosynthesis rate, and greater drought tolerance [22]. CNPs increased water content, efficiency, and photosynthesis, enhancing the growth and yield of tomato plants under drought conditions [21]. Similarly, Shekhawat et al. [19] showed that application of CNPs improved chlorophyll content, protein content, growth, and dry matter accumulation in cowpea plants. CNP application alleviated the damage caused by drought stress on the growth and metabolic and physiological functions of bell pepper plants [22].

Here, foliar application of CNPs effectively boosted physiological activity in both sensitive and tolerant maize hybrids under drought. CNP application to drought-stressed plants further improved water status, water use efficiency, carboxylation efficiency, and photosynthesis (Figure 2, Figure 6 and Figure 7). These metabolic enhancements led to better growth under drought stress conditions, notably in a sensitive maize hybrid (Figure 2, Figure 3 and Figure 5). Many studies confirm CNPs’ role in enhancing plant and crop performance under stressful environmental conditions. The main beneficial effects of CNP application in attenuating the adverse effects of drought and improving drought tolerance include water status regulation [20,21,22], elevated chlorophyll production [19,21,22], better water use efficiency [20], and activation of the antioxidant defense system [19,20,21,22]. CNPs improve WUE by modulating stomatal opening, reducing stomatal conductance, and mitigating water loss through transpiration [33,34,35,36]. Furthermore, CNPs activate several antioxidant enzymes, which protect photosynthetic pigments (chlorophyll) from oxidative stress and increase the plant’s photosynthetic rate (Figure 6A,B). In summary, CNPs induce morphological, physiological, and biochemical changes that heighten drought tolerance, mainly via improved water uptake and organic compound and protein synthesis involved in stress signaling and elimination of reactive oxygen species [34,36].

Overall, our data suggests foliar application of CNPs substantially lessens drought impacts, aiding maize adaptation under stress conditions. These findings highlight the importance of using CNPs as nanofertilizers to stimulate plant growth and improve drought tolerance. Therefore, foliar application of CNPs is a practical tool for managing drought stress in maize crops, which enhances sustainable food production. These findings will provide the basis for further research on the interactions between CNPs and plants and help ensure their safe and sustainable use in global agricultural production.

## 4. Materials and Methods

### 4.1. Plant Material and Treatments

Seeds of two commercial maize hybrids, one drought-tolerant (LG 36745 PRO4) and one drought-sensitive (AG 8088 PRO2), were obtained from the seed market in Chapadão do Sul, Mato Grosso do Sul, Brazil, to examine foliar carbon nanoparticle effects on early growth and physiological traits under stress. The main characteristics of maize seeds and hybrids are shown in Table 2.

Seeds were sterilized with 2% (*w*/*v*) sodium hypochlorite (NaOCl) for 8 min before germination under control (non-stress) and drought stress conditions. Drought stress was induced using polyethylene glycol (PEG-6000) solutions to achieve osmotic potentials of 0, −0.4, and −0.8 MPa. The amount of PEG-6000 required to prepare the solutions with distinct osmotic potentials was calculated using the equation of Michel & Kaufmann [37]:

Ψ_S_ = [−(1.18 × 10^−2^) × C − (1.18 × 10^−4^) × C^2^ + (2.67 ×10^−4^) × C × T + (8.39 × 10^−7^) × C^2^ × T]/10,

where Ψ_S_ is the osmotic potential (MPa), C is the concentration (g L^−1^ of PEG-6000), and T is the temperature (°C). Distilled water (0.00 MPa) served as the control.

Treatments were arranged in a completely randomized design with a 2 × 2 × 3 factorial: two hybrids (drought-tolerant LG 36745 PRO4 and drought-sensitive AG 8088 PRO2), two CNP foliar levels (0 and 1.0 mL L^−1^ of CNP-based nanofertilizer), and three drought levels (0 MPa for control, −0.4 MPa for moderate, and −0.8 MPa for severe), with four replicates.

Carbon nanoparticles (CNPs) were applied as a foliar spray at the V_2_ growth stage (second fully expanded leaf) using Arbolin Biogenesis. This product, commercially known as Arbolina^®^ (Krilltech Nano Agtech, Brasília, Distrito Federal, Brazil), is an organomineral nanofertilizer composed of 10% carbon dots (C-Dots) (*w*:*w*) combined with 4% nitrogen (*w*:*w*). The core of the product consists of carbon-based nanoparticles, which are organic, biodegradable, and biocompatible. The C-dots nanoparticles are spherical with an average size of 3.93 nm (range between 3.5 and 4.2 nm). Their molecular nanostructure has a high active surface area with hydroxyl (-OH), carboxyl (-COOH), and amine (-NH) functional groups, which improve stability, solubility, and the efficiency of foliar nutrient absorption. A 1.0 mL L^−1^ Arbolin Biogenesis solution was applied to maize leaves using a CO_2_-pressurized sprayer at 210 kPa, fitted with three ATR 4.0 cone nozzles and calibrated for 180 L ha^−1^ spray volume. The concentration of 1.0 mL L^−1^ Arbolin Biogenesis applied was based on the nanofertilizer manufacturer’s recommendation (Krilltech Nano Agtech, Brasília, DF, Brazil). This is essential to ensure the effectiveness and safety of the product.

### 4.2. Plant Growth Condition

Seeds were sown 2.0 cm deep in plastic containers (44 × 30 × 7.5 cm) filled with sterilized quartz sand. Sand was sieved (0.05–0.8 mm mesh) and moistened to 70% water retention capacity [38] equivalent to 185 mL kg^−1^ with distilled water (control) or PEG-6000 solutions (stress). Containers were maintained in a laboratory with LED-supplemented light: red (620–630 nm) and blue (455–475 nm) at an 85:15 ratio, 250 ± 80 μmol m^−2^ s^−1^ intensity, 12/12 h photoperiod, and 25.4 ± 2.1 °C temperature for 25 days. Each treatment had four replicates of 50 seeds.

Plants received nutrient solution at 10, 15, and 20 days: 180 mg L^−1^ N, 80 mg L^−1^ P, 300 mg L^−1^ K, 150 mg L^−1^ Ca, 30 mg L^−1^ Mg, 30 mg L^−1^ S, 1.4 mg L^−1^ Fe, 0.4 mg L^−1^ B, 0.4 mg L^−1^ Mn, 0.3 mg L^−1^ Cu, 0.2 mg L^−1^ Zn, 0.01 mg L^−1^ Mo, 0.06 mg L^−1^ Ni, and 0.02 mg L^−1^ Co. Nutrient sources included calcium nitrate, potassium nitrate, magnesium nitrate, monoammonium phosphate, potassium sulfate, magnesium sulfate, iron chelate, manganese chelate, copper chelate, zinc chelate, boric acid, sodium molybdate, nickel sulfate, and cobalt sulfate. Fertilizers were dissolved in the corresponding osmotic solutions for each treatment. Substrate moisture was monitored daily at 2:00 p.m. gravimetrically by weighing containers. Moisture was maintained at 70% sand retention capacity via daily irrigation.

### 4.3. Quantification of Morphological Traits

After 25 days of exposure to drought stress, plant height (PH), leaf area (LA), length of the longest root (LLR), total root system length (TRL), root volume (RV), shoot dry matter (SDM), root dry matter (RDM), and total dry matter (TDM) were measured. The PH and LLR were measured using a ruler. The LA was determined using an automatic leaf area meter (Li-Cor^®^, model LI-3100, Lincoln, NE, USA). The SDM, RDM, and TDM were recorded on an analytical balance (±0.0001 g) after drying in an oven at 85 °C for 48 h. For the determination of TRL and RV, individual plant roots were scanned using an optical scanner (Scanjet 4C/T, HP Development Company, L.P., Barueri, São Paulo, Brazil) at 300 dpi resolution, and the digitized images were analyzed with the WinRhizo program version 3.8-b (Regent Instrument Inc., Quebec, QC, Canada). All measurements were performed on five random plants per replicate.

### 4.4. Quantification of Physiological Traits

After 25 days of exposure to drought stress, the relative water content (RWC) was also calculated based on the weighing of fresh matter (FM) and dry matter (DM) of five randomly chosen seedlings per replicate, using the following equation proposed by Weatherley [39]:

RWC (%) = [(FM − DM)/FM)] × 100.

Photosynthetic rate (*A*), intercellular CO_2_ concentration (Ci), transpiration rate (*E*), and stomatal conductance (*g*_S_) were recorded on the second uppermost fully expanded leaf using a portable infrared gas analyzer (IRGA, Li-6400XT model, LiCor Inc., Lincoln, NE, USA) under standardized light (1000 μmol m^–2^ s^–1^) and CO_2_ conditions (380 μmol mol^–1^). Measurements were taken between 9:00 and 10:00 a.m. in plants exposed to artificial supplemental light radiation. Water use efficiency (WUE) was calculated by the ratio between carbon assimilation (photosynthesis) and water loss through transpiration (WUE = *A*/*E*).

### 4.5. Statistical Analysis

Data were tested for variance homoscedasticity (Levene’s test, *p* > 0.05) and residual normality (Kolmogorov–Smirnov test, *p* > 0.05) before analysis of variance (F-test, α = 0.05). Means were compared using Tukey’s test at α = 0.05. Analyses were conducted with Rbio v. 140 software (Rbio Software, UFV, Viçosa, MG, Brazil).

Canonical variate analysis (CVA) was used to capture the relationships between the set of independent variables (maize hybrid) and dependent variables (plant morphophysiological traits).

## 5. Conclusions

Foliar application of carbon nanoparticles has high potential to mitigate the detrimental impacts of drought on maize plants grown under moderate and severe stress conditions, especially through maintaining plant water status and improving water use efficiency, carboxylation efficiency, photosynthesis rate, and early plant growth under adverse environmental conditions. These findings will provide the scientific basis for future field research on the adoption of management practices to control drought and ensure the development of modern and sustainable agriculture. Our results highlight the key role of applying CNP-based nanofertilizers in improving the physiological metabolism and growth of maize plants. However, these morphophysiological responses of maize plants to CNP application must be proven under field conditions. Furthermore, future research needs to validate the beneficial effects of CNPs on the biochemical and molecular mechanisms of maize plants exposed to drought conditions.

## Figures and Tables

**Table 1:** Summary of analysis of variance for measurements of morphological and physiological traits of maize hybrids (*Zea mays* L.) in response to drought stress levels and foliar application of carbon nanoparticles.

Sources of Variation	Probability > F							
	PH	LA	LLR	TRL	RV	SDM	RDM	TDM
Hybrid (H)	<0.001	<0.001	<0.001	<0.001	<0.001	0.012	<0.001	<0.001
Drought (D)	<0.001	<0.001	<0.001	<0.001	<0.001	<0.001	0.002	<0.001
Nanoparticles (CNPs)	<0.001	<0.001	<0.001	<0.001	<0.001	<0.001	<0.001	<0.001
H × D	<0.001	<0.001	<0.001	0.001	<0.001	0.065	<0.001	0.003
H × CNPs	0.367	0.054	0.040	<0.001	<0.001	<0.001	<0.001	<0.001
D × CNPs	<0.001	0.048	<0.001	0.072	0.018	0.036	0.550	0.112
H × D × CNPs	0.476	0.849	0.157	0.191	0.308	<0.001	0.134	<0.001
CV (%)	4.64	4.88	4.32	6.30	5.81	4.97	5.45	4.43
	RWC	*A*	Ci	*E*	*g*_S_	WUE	*A*/Ci	
Hybrid (H)	<0.001	<0.001	<0.001	<0.001	<0.001	<0.001	<0.001	
Drought (D)	<0.001	<0.001	<0.001	<0.001	<0.001	<0.001	<0.001	
Nanoparticles (CNPs)	<0.001	<0.001	<0.001	<0.001	0.067	<0.001	<0.001	
H × D	<0.001	<0.001	<0.001	<0.001	<0.001	0.035	0.015	
H × CNPs	0.018	0.028	0.044	0.019	0.035	0.048	0.004	
D × CNPs	<0.001	0.042	0.038	0.037	0.068	0.132	0.146	
H × D × CNPs	0.012	0.214	0.359	0.403	0.609	0.544	0.799	
CV (%)	3.79	6.47	4.15	3.36	8.70	4.58	9.03	

PH: plant height. LA: leaf area. LLR: length of the longest root. TRL: total root system length. RV: root volume. SDM: shoot dry matter. RDM: root dry matter. TDM: total dry matter. RWC: relative water content. *A*: photosynthetic rate. Ci: intercellular CO_2_ concentration. *E*: transpiration rate. *g*_S_: stomatal conductance. WUE: water use efficiency. *A*/Ci: carboxylation efficiency.

**Table 2:** Characteristics of maize hybrids (*Zea mays* L.) grown under non-stressful and drought stress conditions.

Maize Hybrid	Origin	Maturation Cycle (Days)	Yield Potential	1000-SW (g)	GR (%)	Drought Stress Response ^†^
LG 36745 PRO4	LG^®^ Seeds ^1^	136	High	330	98	Tolerant
AG 8088 PRO2	Agroceres^®^ Seeds ^2^	135	High	380	94	Sensitive

^1^ Limagrain Field Seeds Brasil, Curitiba, Paraná, Brazil. ^2^ Sementes Agroceres, Goiânia, Goiás, BRA. 1000-SW: 1000-seed weight. GR: germination rate. ^†^ Reference: Vilas Boas et al. [3].

## Data Availability

Data will be made available on request.
